# Timing and Completeness of Trial Results Posted at ClinicalTrials.gov and Published in Journals

**DOI:** 10.1371/journal.pmed.1001566

**Published:** 2013-12-03

**Authors:** Carolina Riveros, Agnes Dechartres, Elodie Perrodeau, Romana Haneef, Isabelle Boutron, Philippe Ravaud

**Affiliations:** 1 INSERM U738, Paris, France; 2 Université Paris Descartes—Sorbonne Paris Cité, Paris, France; 3 Centre d'Épidémiologie Clinique, Hôpital Hôtel-Dieu, Assistance Publique-Hôpitaux de Paris, Paris, France; 4 French Cochrane Centre, Paris, France; 5 Mailman School of Public Health, Columbia University, New York, New York, United States of America; Center for Clinical Trials, United States of America

## Abstract

**Background:**

The US Food and Drug Administration Amendments Act requires results from clinical trials of Food and Drug Administration–approved drugs to be posted at ClinicalTrials.gov within 1 y after trial completion. We compared the timing and completeness of results of drug trials posted at ClinicalTrials.gov and published in journals.

**Methods and Findings:**

We searched ClinicalTrials.gov on March 27, 2012, for randomized controlled trials of drugs with posted results. For a random sample of these trials, we searched PubMed for corresponding publications. Data were extracted independently from ClinicalTrials.gov and from the published articles for trials with results both posted and published. We assessed the time to first public posting or publishing of results and compared the completeness of results posted at ClinicalTrials.gov versus published in journal articles. Completeness was defined as the reporting of all key elements, according to three experts, for the flow of participants, efficacy results, adverse events, and serious adverse events (e.g., for adverse events, reporting of the number of adverse events per arm, without restriction to statistically significant differences between arms for all randomized patients or for those who received at least one treatment dose).

From the 600 trials with results posted at ClinicalTrials.gov, we randomly sampled 50% (*n* = 297) had no corresponding published article. For trials with both posted and published results (*n* = 202), the median time between primary completion date and first results publicly posted was 19 mo (first quartile = 14, third quartile = 30 mo), and the median time between primary completion date and journal publication was 21 mo (first quartile = 14, third quartile = 28 mo). Reporting was significantly more complete at ClinicalTrials.gov than in the published article for the flow of participants (64% versus 48% of trials, *p*<0.001), efficacy results (79% versus 69%, *p* = 0.02), adverse events (73% versus 45%, *p*<0.001), and serious adverse events (99% versus 63%, *p*<0.001).

The main study limitation was that we considered only the publication describing the results for the primary outcomes.

**Conclusions:**

Our results highlight the need to search ClinicalTrials.gov for both unpublished and published trials. Trial results, especially serious adverse events, are more completely reported at ClinicalTrials.gov than in the published article.

*Please see later in the article for the Editors' Summary*

## Abstract 2

Agnes Dechartres and colleagues searched ClinicalTrials.gov for completed drug RCTs with results reported and then searched for corresponding studies in PubMed to evaluate timeliness and completeness of reporting.

*Please see later in the article for the Editors' Summary*

## Editors' Summary

**Background:**

When patients consult a doctor, they expect to be recommended what their doctor believes is the most effective treatment with the fewest adverse effects. To determine which treatment to recommend, clinicians rely on sources that include research studies. Among studies, the best evidence is generally agreed to come from systematic reviews and randomized controlled clinical trials (RCTs), studies that test the efficacy and safety of medical interventions by comparing clinical outcomes in groups of patients randomly chosen to receive different interventions. Decision-making based on the best available evidence is called evidence-based medicine. However, evidence-based medicine can only guide clinicians if trial results are published in a timely and complete manner. Unfortunately, underreporting of trials is common. For example, an RCT in which a new drug performs better than existing drugs is more likely to be published than one in which the new drug performs badly or has unwanted adverse effects (publication bias). There can also be a delay in publishing the results of negative trials (time-lag bias) or a failure to publish complete results for all the prespecified outcomes of a trial (reporting bias). All three types of bias threaten informed medical decision-making and the health of patients.

**Why Was This Study Done?:**

One initiative that aims to prevent these biases was included in the 2007 US Food and Drug Administration Amendments Act (FDAAA). The Food and Drug Administration (FDA) is responsible for approving drugs and devices that are marketed in the US. The FDAAA requires that results from clinical trials of FDA-approved drugs and devices conducted in the United States be made publicly available at ClinicalTrials.gov within one year of trial completion. ClinicalTrials.gov—a web-based registry that includes US and international clinical trials—was established in 2000 in response to the 1997 FDA Modernization Act, which required mandatory registration of trial titles and designs and of the conditions and interventions under study. The FDAAA expanded these mandatory requirements by requiring researchers studying FDA-approved drugs and devices to report additional information such as the baseline characteristics of the participants in each arm of the trial and the results of primary and secondary outcome measures (the effects of the intervention on predefined clinical measurements) and their statistical significance (an indication of whether differences in outcomes might have happened by chance). Researchers of other trials registered in ClinicalTrials.gov are welcome to post trial results as well. Here, the researchers compare the timing and completeness (i.e., whether all relevant information was fully reported) of results of drug trials posted at ClinicalTrials.gov with those published in medical journals.

**What Did the Researchers Do and Find?:**

The researchers searched ClinicalTrials.gov for reports of completed phase III and IV (late-stage) RCTs of drugs with posted results. For a random sample of 600 eligible trials, they searched PubMed (a database of biomedical publications) for corresponding publications. Only 50% of trials with results posted at ClinicalTrials.gov had a matching published article. For 202 trials with both posted and published results, the researchers compared the timing and completeness of the results posted at ClinicalTrials.gov and of results reported in the corresponding journal publication. The median time between the study completion date and the first results being publicly posted at ClinicalTrials.gov was 19 months, whereas the time between completion and publication in a journal was 21 months. The flow of participants through trials was completely reported in 64% of the ClinicalTrials.gov postings but in only 48% of the corresponding publications. Results for the primary outcome measure were completely reported in 79% and 69% of the ClinicalTrials.gov postings and corresponding publications, respectively. Finally, adverse events were completely reported in 73% of the ClinicalTrials.gov postings but in only 45% of the corresponding publications, and serious adverse events were reported in 99% and 63% of the ClinicalTrials.gov postings and corresponding publications, respectively.

**What Do These Findings Mean?:**

These findings suggest that the reporting of trial results is significantly more complete at ClinicalTrials.gov than in published journal articles reporting the main trial results. Certain aspects of this study may affect the accuracy of this conclusion. For example, the researchers compared the results posted at ClinicalTrials.gov only with the results in the publication that described the primary outcome of each trial, even though some trials had multiple publications. Importantly, these findings suggest that, to enable patients and physicians to make informed treatment decisions, experts undertaking assessments of drugs should consider seeking efficacy and safety data posted at ClinicalTrials.gov, both for trials whose results are not published yet and for trials whose results are published. Moreover, they suggest that the use of templates to guide standardized reporting of trial results in journals and broader mandatory posting of results may help to improve the reporting and transparency of clinical trials and, consequently, the evidence available to inform treatment of patients.

**Additional Information:**

Please access these websites via the online version of this summary at http://dx.doi.org/10.1371/journal.pmed.1001566.

- Wikipedia has pages on evidence-based medicine and on publication bias (note: Wikipedia is a free online encyclopedia that anyone can edit; available in several languages)
- The US Food and Drug Administration provides information about drug approval in the US for consumers and health-care professionals, plus detailed information on the 2007 Food and Drug Administration Amendments Act
- ClinicalTrials.gov provides information about the US National Institutes of Health clinical trial registry, including background information about clinical trials, and a fact sheet detailing the requirements of the 2007 Food and Drug Administration Amendments Act
- *PLOS Medicine* recently launched a Reporting Guidelines Collection, an open access collection of reporting guidelines, commentary, and related research on guidelines from across PLOS journals that aims to help advance the efficiency, effectiveness, and equitability of the dissemination of biomedical information; a 2008 PLOS Medicine editorial discusses the 2007 Food and Drug Administration Amendments Act

## Introduction

Without accessible and usable reports, research fails to help patients and their clinicians [1]. Over the past decades, underreporting of trial results has been increasingly acknowledged as one of the main causes of waste of research [2]–[5], contributing to biased evidence, with serious consequences for clinical practice, research, and, ultimately, patients [1]. This waste of research can occur at different stages: (1) failure to publish results of some studies, particularly those with negative results, “publication bias” [6]–[8]; (2) delay in publishing results of negative studies [9], “time-lag bias” [10]; and (3) failure to publish complete results for all prespecified outcomes, “reporting bias” [10]–[14]. Among published studies, some results may be incompletely reported and therefore cannot be included in a meta-analysis. This is the case, for example, when the difference in means between treatments is reported but not a measure of precision.

To overcome these issues, the 2007 US Food and Drug Administration Amendments Act (FDAAA) requires that the results from clinical trials of Food and Drug Administration–approved drugs and devices conducted in the United States must be made publicly available at ClinicalTrials.gov within 1 y of the completion of the trial, whether the results are published or not [15]–[18]. This US public law requires a “table of the demographic and baseline data collected overall and for each arm of the clinical trial to describe the patients who participated in the clinical trial … [and] a table of values for each of the primary and secondary outcome measures for each arm of the clinical trial, including the results of scientifically appropriate tests of the statistical significance of such outcome measures.” Researchers of other trials registered in ClinicalTrials.gov are welcome to post trial results as well. In our study, we aimed to compare the timing and completeness (i.e., whether all relevant information was fully reported) of results publicly posted at ClinicalTrials.gov and in published articles for trials of drug interventions.

## Methods

We identified trials with results posted on ClinicalTrials.gov and their corresponding full-text publications in journals.

### Search for Trials with Results Posted at ClinicalTrials.gov

We searched ClinicalTrials.gov on March 27, 2012, using the following keywords: “Closed study” in the Recruitment field, “With results” for Study Results, “Interventional studies” for Study Type, and “Phase III and IV” for Phase. We selected completed phase III or IV randomized controlled trials listing only drugs as intervention type. We excluded trials comparing a drug to a device. We excluded phase II/III trials, considering them to be phase II trials. Of all eligible trials (*n* = 1,592), we selected a random sample of 600 trials for which to search for full-text publications.

### Search for Publication of Results in Journals

Whenever possible, we used the link within ClinicalTrials.gov to identify the published article. We also systematically searched MEDLINE via PubMed by using the ClinicalTrials.gov identification number (NCT number). If no publication was identified, we searched MEDLINE via PubMed again by using keywords for drug names and the condition studied. The articles identified through the search had to match the corresponding trial in terms of the information registered at ClinicalTrials.gov (i.e., same objective, same sample size, same primary outcome, same location, same responsible party, same trial phase, and same sponsor) and had to present results for the primary outcome. A second reviewer checked the matching between ClinicalTrials.gov and the published article. All disagreements were resolved by discussion between the two reviewers.

In order to compare the reporting between the ClinicalTrials.gov report and the published article, we excluded trials for which results were published in a journal that could not be retrieved or were published in a language other than English, French, or Spanish. We also excluded single-arm studies because they lacked a control group, as well as studies with four or more arms, for practical reasons.

### Data Extraction

We collected the following information from ClinicalTrials.gov for the random sample of 600 trials with results posted at ClinicalTrials.gov:

1. General characteristics of the trial: primary funding sources (extracted from Study Sponsor at ClinicalTrials.gov), medical specialty (extracted from Conditions at ClinicalTrials.gov), and countries where the trial was conducted (extracted from Location Countries at ClinicalTrials.gov). We also collected trial primary completion date (defined as the date of final collection of data for the primary outcome) and date when results were first publicly posted—this date was extracted from the archive record and differs from the date on which results were first received, which is available under Study Results at ClinicalTrials.gov. The difference between these two dates is related to production and the vetting of the results by the US National Institutes of Health.
2. Design of the trial: we noted whether the trial was a phase III or IV trial. We recorded whether it was a parallel or crossover trial.
3. Interventions: details concerning interventions for experimental and control groups were extracted from Study Arm(s) at ClinicalTrials.gov.

Then, for all trials with both results posted and published, we collected the following information independently from ClinicalTrials.gov and from the published article:

1. Flow of participants in the trial: reporting of the flow of participants, including number of participants assessed for eligibility, number of participants randomized overall and per arm, number of participants who received the intervention per arm and whether the reasons for not receiving the intervention were specified, number of patients lost to follow-up and those who discontinued the intervention and whether the reasons for discontinuation were given, and number of participants analyzed per arm and whether the reasons for excluding participants from the analysis were reported.
2. Efficacy results: for the primary outcome posted at ClinicalTrials.gov, we extracted data from ClinicalTrials.gov for this outcome. If several primary outcomes were posted at ClinicalTrials.gov, we focused on the first registered. When we extracted efficacy results in the published article, we extracted data for this outcome whether it was reported as primary or secondary. If the outcome was not reported at all, we considered the data to be missing. For all types of outcomes, we collected whether numbers of patients analyzed per arm were reported. For binary outcomes, we collected whether the number of events per arm was reported. For continuous outcomes, we collected whether (1) mean (± standard deviation [SD]) or (2) median (interquartile) was reported or (3) neither of these was reported. For time-to-event outcomes, we collected whether the results of the log-rank test or Cox proportional hazard model were reported.
3. Adverse events: we noted whether adverse events were reported and whether they were reported by number per arm. We collected whether all adverse events were reported or only common events were reported or those with statistically significant differences between arms. We noted whether reporting of adverse events concerned all randomized participants or only those who received at least one treatment dose. We also collected whether withdrawals due to adverse events were reported.
4. Serious adverse events: we collected whether serious adverse events were reported, were reported per arm, and were reported with numerical data.

All data were extracted in duplicate by two reviewers in data collection forms. All disagreements were resolved by discussion to reach a consensus, including intervention of a third reviewer in case of discrepancies.

We also extracted the characteristics of the publication (i.e., the journal in which the article was published, the date of online publication, the type of journal [general medical, medical specialty], and whether the NCT number was reported in the published article).

### Assessment of the Completeness of Reporting

Three experts in clinical epidemiology reached consensus on the main elements that needed to be reported for each of the following domains: the flow of participants during the trial, efficacy results, adverse events, and serious adverse events, on the basis of data required to perform meta-analyses. The reporting was considered complete for each domain if all of the included elements in Box 1 were reported and incomplete if one or more elements were missing from the items listed in Box 1.

**Box 1. Definition of Completeness of Reporting**

The reporting was considered complete for each domain if all of the included elements were reported and incomplete if one or more elements were missing:

**Flow of participants**

Number of patients randomized per arm and
Number of patients lost to follow-up per arm and
Number of patients analyzed per arm

**Efficacy results**

**For binary outcomes:**
  Number of events per arm and
  Number of patients analyzed per arm
**For continuous outcomes:**
  Mean or median per arm and SD or SE or 95% CI or Q1–Q3 or
  Effect size (difference in means or standardized mean difference) with 95% CI
**For time-to-event outcomes:**
  Hazard ratio with 95% CI

**Adverse events**

Number of adverse events per arm without restriction to statistically significant differences between arms for all randomized participants or for those who received at least one treatment dose

**Serious adverse events**

Number of serious adverse events per arm

In a second step, we compared the number of elements reported at ClinicalTrials.gov and in the published article for the flow of participants, efficacy results, adverse events, and serious adverse events. We assessed the number of pairs (with percentage) for which ClinicalTrials.gov provided more information (higher number of elements reported), similar information (same number of elements reported), and less information (lower number of elements reported) as compared with the published article for the flow of participants, efficacy results, adverse events, and serious adverse events as defined above.

### Statistical Analyses

Inter-rater agreement between the two reviewers was assessed by the Kappa coefficient (95% CI). Descriptive analyses of trial characteristics included numbers and percentages. Time from trial primary completion date to the date of posting of results at ClinicalTrials.gov or online publication in journals was described with the Kaplan and Meier method for trials with results both posted at ClinicalTrials.gov and published.

We compared results posted at ClinicalTrials.gov and in the published article for completeness of reporting using McNemar's test of equality of paired proportions. We tested the interaction between completeness of reporting at ClinicalTrials.gov and in the published article and the following trial characteristics: type of journal (general versus specialty), type of control (active versus placebo or no treatment), source of funding (academic, industry, both), and study design (parallel arms versus crossover) using a generalized estimation equation (GEE) model to take into account the correlation between the paired observations.

All tests were two-tailed, and *p*<0.05 was considered statistically significant. Analyses were conducted using R version 2.15.1 [19].

## Results

**Figure 1:**
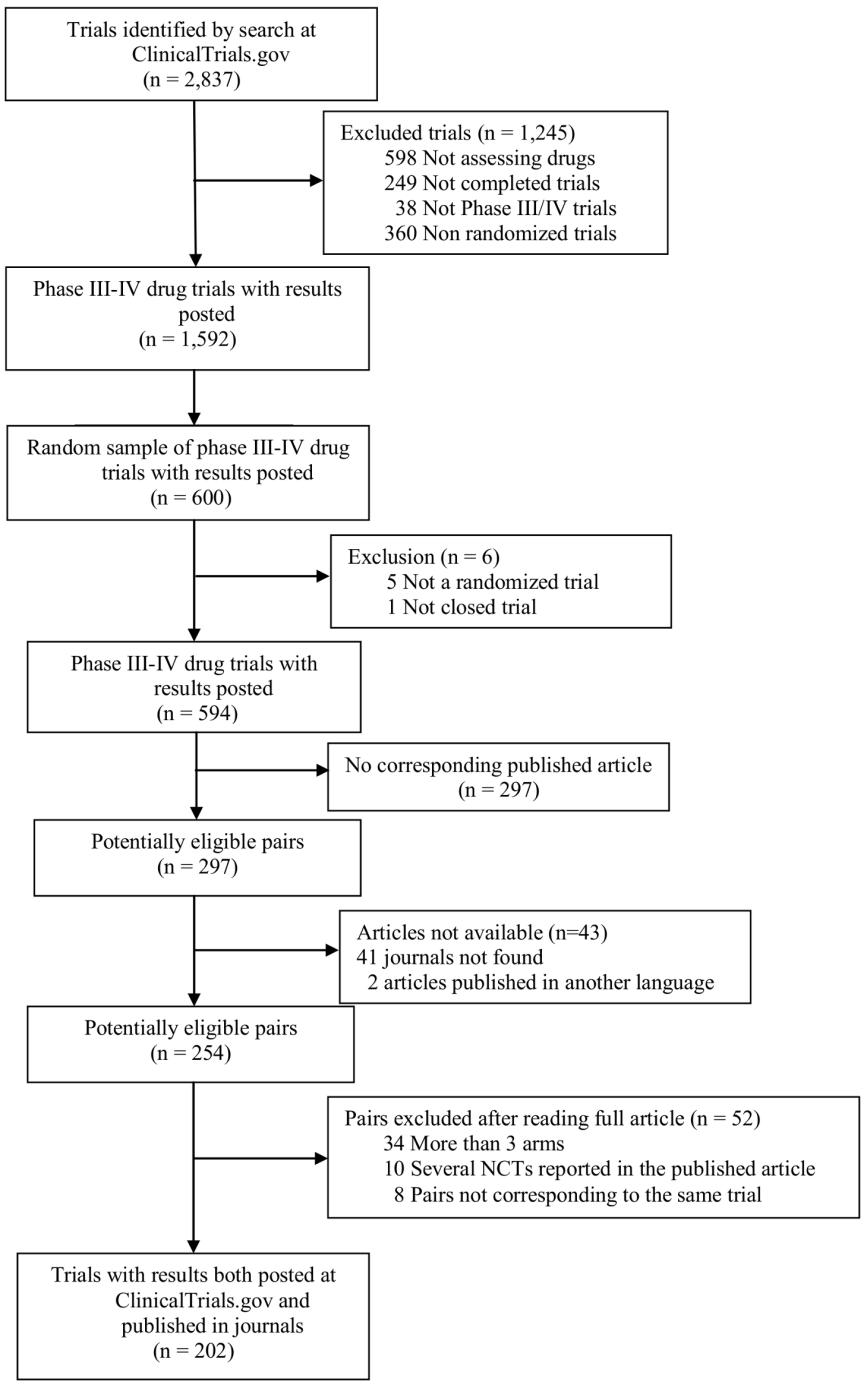
Flow diagram of selection of relevant trials. NCTs, NCT numbers.

Figure 1 describes the selection of trials. Briefly, from the 2,837 trials retrieved by a search of ClinicalTrials.gov on March 27, 2012, we identified 1,592 completed phase III or IV randomized drug trials with results posted at ClinicalTrials.gov. We selected a random sample of 600 trials to search for corresponding publications.

### Time to Posting Results at ClinicalTrials.gov and Publication in Journals

**Figure 2:**
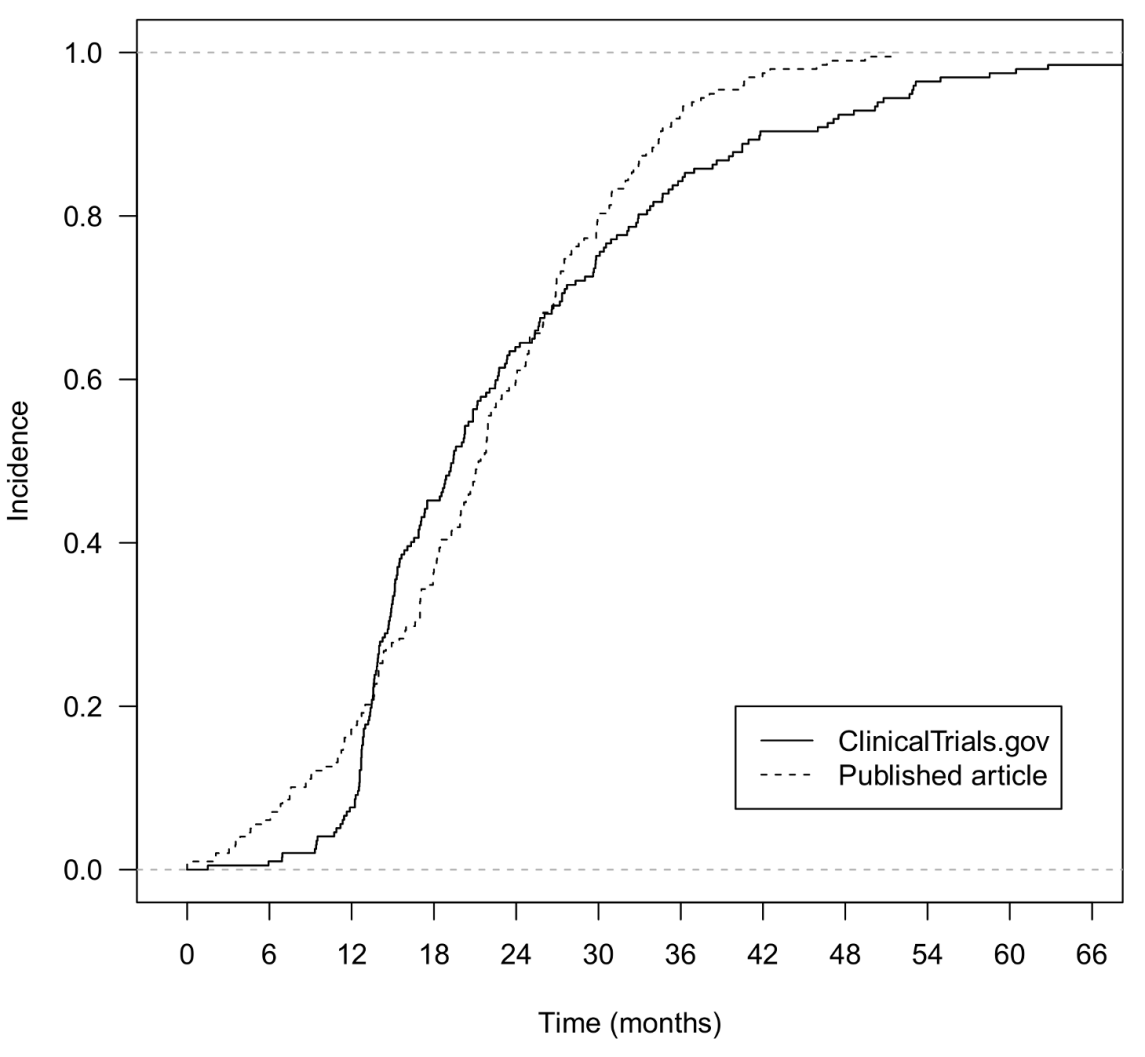
Comparison of time from primary completion date of the trial to posting of results at ClinicalTrials.gov and to online publication in journals for trials with both posted and published results.

Of the 600 trials with results posted at ClinicalTrials.gov that were randomly selected for a search for corresponding publications, we excluded five that were not randomized controlled trials, and one that was still enrolling patients. From the remaining 594 trials, 297 (50%) had no corresponding published article. The median year of completion of trials with no corresponding article was 2009 (first quartile [Q1] = 2008, third quartile [Q3] = 2009). We included 202 pairs of reports for publicly posted and published results. In total, 18 of the 202 trials (9%) had multiple publications (from two to 14). These reports concerned protocols or preliminary results such as baseline results (*n* = 6), interim analyses (*n* = 3), long-term outcomes (*n* = 15), other outcomes (*n* = 19), and/or other results (*n* = 1). None of the eliminated reports contained additional safety data for the same time frame as the selected report. Two of the eliminated reports contained additional safety results for longer-term follow-up. The median time between primary completion date and results first being publicly posted at ClinicalTrials.gov was 19 mo (Q1 = 14, Q3 = 30 mo); the median time between primary completion date and publication in journals was 21 mo (Q1 = 14, Q3 = 28 mo) (Figure 2).

### Characteristics of Trials with Both Posted and Published Results

Inter-rater agreement between the 2 reviewers was good overall, with median Kappa coefficient 0.80 (range 0.47–0.95) for qualitative variables and 0.98 (0.91–0.99) for quantitative variables.

For the 202 trials with both posted and published results, 69% (139/202) were phase III trials and 31% (63/202) were phase IV trials (Table 1). The primary source of funding was industry for 85% (173/202) of trials, academic for 10% (20/202), and both for 5% (9/202). In total, 75% (152/202) of trials had at least one site in the United States; for 68% (138/202), results were published in specialty medical journals, and for 32% (64/202) in general journals. For 79% (160/202) of trials, the NCT number was reported in the published article.

**Table 1:** Characteristics of the random sample of 600 trials with results posted at ClinicalTrials.gov for which publications were sought.

Item	Characteristic	Number (Percent) of Trials with Characteristic	
		Sample of Trials with Results Posted at ClinicalTrials.gov (*n* = 600)	Sample of Trials with Results Both Posted and Published (*n* = 202)
**Study phase**			
	Phase III	392 (65)	139 (69)
	Phase IV	208 (35)	63 (31)
**Study design**			
	Parallel arms	550 (92)	191 (95)
	Crossover	50 (8)	11 (5)
**Number of arms**			
	Two	412 (69)	158 (78)
	Three	113 (19)	44 (22)
	Other	75 (12)	0 (0)
**Type of control treatment**			
	Active treatment	356 (59)	105 (52)
	Placebo	223 (37)	95 (47)
	No treatment	21 (4)	2 (1)
**Primary funding source**			
	Industry	509 (85)	173 (85)
	Academic	60 (10)	20 (10)
	Academic and industry	31 (5)	9 (5)
**Medical specialty**			
	Pulmonary	58 (10)	27 (14)
	Neurology	37 (7)	21 (10)
	Endocrinology	65 (11)	21 (10)
	Cardiology	53 (9)	20 (10)
	Rheumatology	35 (6)	19 (9)
	Immunology	43 (7)	18 (9)
	Oncology	25 (4)	14 (7)
	Others	284 (47)	62 (31)
**Study location**			
	At least one site in the US	423 (70)	152 (75)
	No site in the US	177 (30)	50 (25)
**Type of journal**			
	Specialty		138 (68)
	General		64 (32)
**NCT number reported in the published article**			
	Yes		160 (79)
	No		42 (21)

### Reporting of Flow of Participants

For the 202 trials with both publicly posted and published results, the number of patients assessed for eligibility was reported for 1% (2/202) of trials at ClinicalTrials.gov and for 65% (131/202) in the published article (Table 2). The number of patients allocated to the intervention group was reported for 99% (200/202) of trials at ClinicalTrials.gov and for 95% (192/202) in the published article. The number of patients lost to follow-up per group was reported for 66% (133/202) of trials at ClinicalTrials.gov and for 53% (108/202) in the published article. The number of patients analyzed was reported for 96% (193/202) of trials at ClinicalTrials.gov and for 88% (177/202) in the published article. The reasons for participant exclusion from the analysis were reported for only 8% of trials (16/202) at ClinicalTrials.gov and 7% (14/202) of published articles.

**Table 2:** Reporting of items concerning the flow of participants during the trial at ClinicalTrials.gov and in published articles.

Category	Item	Number (Percent) of Trials with Item Reported at ClinicalTrials.gov (*n* = 202)	Number (Percent) of Trials with Item Reported in Published Article (*n* = 202)
**Enrollment**			
	Number of participants assessed for eligibility (overall)	2 (1)	131 (65)
	Number of participants excluded (overall)	2 (1)	95 (47)
	Reasons given for excluding participants	0 (0)	70 (74)
	Number of participants randomized (overall)	5 (2)	181 (90)
**Allocation**			
	Number of participants allocated to intervention (per group)	200 (99)	192 (95)
	Number of participants who received allocated intervention (per group)	28 (14)	80 (40)
	Number of participants who did not receive allocated intervention (per group)	10 (5)	40 (21)
	Reasons given for why participants did not receive intervention (per group)	2 (20)	12 (30)
**Follow-up**			
	Number of participants lost to follow-up (per group)	133 (66)	108 (53)
	Number of participants who discontinued intervention (per group)	183 (91)	140 (69)
	Reported reasons for discontinuation (per group)	124 (68)	110 (79)
**Analysis**			
	Number of participants analyzed (per group)	193 (96)	177 (88)
	Reported reasons for exclusion of participants from analysis (per group)	16 (8)	14 (7)

### Reporting of Efficacy Results

For trials with a binary outcome (*n* = 73), the number of patients analyzed was reported for 97% (71/73) of trials at ClinicalTrials.gov and for 89% (65/73) in the published article (Table 3). The number of events was reported for 55% (40/73) of trials at ClinicalTrials.gov and for 66% (48/73) in the published article. For trials with a continuous outcome (*n* = 107), the mean or median was reported for 100% (107/107) of trials at ClinicalTrials.gov and for 90% (96/107) in the published article (Table 3). Dispersion was reported for 96% (103/107) of trials at ClinicalTrials.gov and for 64% (69/107) in the published article. For trials with a time-to-event outcome (*n* = 22), the median time to event was reported for 50% (11/22) of trials at ClinicalTrials.gov and for 41% (9/22) in the published article (Table 3). The number of events was reported for 32% (7/22) of trials at ClinicalTrials.gov and for 32% (7/22) in the published article. The hazard ratio with 95% CI was reported for 68% (15/22) of trials at ClinicalTrials.gov and for 73% (16/22) in the published article.

**Table 3:** Reporting of efficacy results at ClinicalTrials.gov and in published articles.

Outcome	Efficacy Result Item	Number (Percent) of Trials with Item Reported at ClinicalTrials.gov (*n* = 202)	Number (Percent) of Trials with Item Reported in Published Article (*n* = 202)
**Binary primary outcome**	**Number of trials**	**73 (36)**	**73 (36)**
	Number of patients analyzed	71 (97)	65 (89)
	Number of events	40 (55)	48 (66)
**Continuous primary outcome**	**Number of trials**	**107 (53)**	**107 (53)**
	Number of patients analyzed	107 (100)	91 (85)
	Final value	31 (29)	24 (22)
	Change from baseline	76 (71)	83 (78)
	Mean or median	107 (100)	96 (90)
	SD, SE, 95% CI, Q1–Q3	103 (96)	69 (64)
	Effect size with 95% CI	42 (39)	47 (44)
**Time-to-event primary outcome**	**Number of trials**	**22 (11)**	**22 (11)**
	Number of patients analyzed	22 (100)	19 (86)
	Number of events	7 (32)	7 (32)
	Median	11 (50)	9 (41)
	Hazard ratio with 95% CI	15 (68)	16 (73)

The comparison of efficacy results between ClinicalTrials.gov and the published article could not be performed in 96/202 (48%) trials:

1. For 42/73 (58%) trials with binary outcomes the comparison could not be made because of incomplete reporting of the number of events. This information was missing in 33 (79%) trials at ClinicalTrials.gov, 25 (60%) in the published article, and 16 (38%) in both data sources. For the 31 trials reporting this information both at ClinicalTrials.gov and in the published article, the numbers of events were the same for 27 (87%).
2. For 45/107 (42%) trials with continuous outcomes, the comparison could not be made: nine (20%) because of different types of analysis (e.g., the final-value mean [SD] was reported at ClinicalTrials.gov, experimental arm, 1.12 [0.54], and control arm, 1.14 [0.55], while the change-from-baseline mean [95% CI] was reported in the published article, experimental arm, 0.59 [0.47 to 0.71], and control arm, 0.54 [0.41 to 0.67]), and 36 (80%) because of incomplete or different reporting between the two sources (e.g., different rounding used; SD reported in one source and standard error [SE] reported in the other).
3. For 9/22 (41%) trials with time-to-event outcomes the comparison could not be made because of incomplete reporting of hazard ratio (95% CI). This information was missing in seven (78%) trials at ClinicalTrials.gov, six (67%) in the published article, and four (44%) in both data sources.

### Reporting of Adverse Events

For the 202 pairs of trial reports, the population for analysis corresponded to all randomized participants for 57% (115/202) of trials at ClinicalTrials.gov and 36% (72/202) of published articles (Table 4). The total number of adverse events was reported for 96% (194/202) of trials at ClinicalTrials.gov and for 63% (128/202) in the published article. All adverse events per arm were reported for 13% (26/202) of trials at ClinicalTrials.gov and for 5% (10/202) in the published article. Otherwise, reporting was restricted to the most common events for 99% (174/176) of trials at ClinicalTrials.gov and for 44% (85/192) in the published article, or to statistically significant events for 15% (29/192) of trials in the published article. Withdrawals due to adverse events were reported for 80% (161/202) of trials in ClinicalTrials.gov and for 76% (153/202) in the published article. There was some mention of serious adverse events for 99% (200/202) of trials at ClinicalTrials.gov and for 71% (144/202) in the published article, and all serious adverse events were reported per arm for 99% (199/202) and 63% (127/202), respectively.

**Table 4:** Reporting of adverse events at ClinicalTrials.gov and in published articles.

Category	Adverse Event Item	Number (Percent) of Trials with Item Reported at ClinicalTrials.gov (*n* = 202)	Number (Percent) of Trials with Item Reported in Published Article (*n* = 202)
**Adverse events**	**Population**		
	All randomized participants	115 (57)	72 (36)
	If no, patients who received at least one dose of treatment	34 (39)	57 (44)
	**Total number of adverse events**	194 (96)	128 (63)
	**Reporting of adverse events**		
	Details of all adverse events per arm	26 (13)	10 (5)
	If no, restriction to most common events (e.g., occurring in ≥5%)	174 (99)	85 (44)
	If no, restriction to statistically significant events	0 (0)	29 (15)
	**Withdrawals due to adverse events**	161 (80)	153 (76)
**Serious adverse events**	**Population**		
	All randomized participants	115 (57)	72 (36)
	If no, patients who received at least one dose of treatment	34 (39)	57 (44)
	**Reporting of serious adverse events**	200 (99)	144 (71)
	**Details of all serious adverse events per arm**	199 (99)	127 (63)

### Completeness of Reporting

For the 202 pairs of trial reports, the proportion of trials with complete reporting was significantly higher at ClinicalTrials.gov than in the published article for the flow of participants (64% [129/202] versus 48% [96/202] of trials, *p*<0.001), efficacy results (79% [159/202] versus 69% [140/202], *p* = 0.02), adverse events (73% [147/202] versus 45% [91/202], *p*<0.001), and serious adverse events (99% [199/202] versus 63% [127/202], *p*<0.001) (Table 5).

**Table 5:** Completeness of reporting for the flow of participants during the trial, efficacy results, adverse events, and serious adverse events.

Domain	Definition of Completeness	Number (Percent) of Trials with Complete Reporting at ClinicalTrials.gov (*n* = 202)	Number (Percent) of Trials with Complete Reporting in Published Article (*n* = 202)	*p*-Value
Flow of participants	Reporting of:- Number of patients randomized per arm and- Number of patients lost to follow-up per arm and- Number of patients analyzed per arm	129 (64)	96 (48)	<0.001
Efficacy results	Reporting of:- For binary data: number of events and analyzed patients per arm- For continuous data: mean or median per arm and SD or SE or 95% CI or Q1–Q3 per arm, or effect size (difference in means or standardized mean difference) with 95% CI- For time-to-event data: hazard ratio and 95% CI	159 (79)	140 (69)	0.02
Adverse events	Reporting of:- Number of adverse events per arm, without restriction to statistically significant differences between arms, for all randomized patients or for those who received at least one treatment dose	147 (73)	91 (45)	<0.001
Serious adverse events	Reporting of:- Number of serious adverse events per arm	199 (99)	127 (63)	<0.001

We found statistically significant interactions between completeness of reporting of adverse events and type of journal (74% and 38%, respectively, in ClinicalTrials.gov and in the published article for trials published in a specialty journal versus 70% and 59%, respectively, for trials published in a general journal, *p* for interaction = 0.015) as well as source of funding (75% and 5%, respectively, in ClinicalTrials.gov and in the published article for trials with academic funding; 73% and 50%, respectively, for trials with industry funding; and 56% and 33%, respectively, for trials with academic and industry funding, *p* for interaction = 0.01).

There was more information reported at ClinicalTrials.gov than in the published article for 29% (59/202) of trials for the flow of participants, 21% (42/202) for efficacy results, 40% (80/202) for adverse events, and 36% (73/202) for serious adverse events. There was less information in ClinicalTrials.gov than in the published article for 10% (21/202) of trials for the flow of participants, 10% (21/202) for efficacy results, 12% (24/202) for adverse events, and 1% (1/202) for serious adverse events of trials (Table 6).

**Table 6:** Comparison of information reported at ClinicalTrials.gov versus in the published article.

Domain	Definition of Completeness	Number (Percent) of Trials with More Information at ClinicalTrials.gov than in the Published Article	Number (Percent) of Trials with Similar Information at ClinicalTrials.gov and in the Published Article	Number (Percent) of Trials with Less Information at ClinicalTrials.gov than in the Published Article
Flow of participants	Reporting of:- Number of patients randomized per arm and- Number of patients lost to follow-up per arm and- Number of patients analyzed per arm	59/202 (29)	122/202 (60)	21/202 (10)
Efficacy results	Reporting of:- For binary data: number of events and analyzed patients per arm- For continuous data: mean or median per arm and SD or SE or 95% CI or Q1–Q3 per arm, or effect size (difference in means or standardized mean difference) with 95% CI- For time-to-event data: hazard ratio and 95% CI	42/202 (21)	139/202 (69)	21/202 (10)
Adverse events	Reporting of- No. of adverse events per arm, without restriction to statistically significant differences between arms, for all randomized patients or for those who received at least one treatment dose	80/202 (40)	98/202 (48)	24/202 (12)
Serious adverse events	Reporting of:- No. of serious adverse events per arm	73/202 (36)	128/202 (63)	1/202 (1)

## Discussion

To our knowledge, this is the first study comparing the timing and completeness of trial results publicly posted at ClinicalTrials.gov and published in articles. Our study is based on a random sample of 600 drug trials with results posted at ClinicalTrials.gov for which we searched for corresponding publications in PubMed. For half of the trials, results were not yet published. For trials with results both posted at ClinicalTrials.gov and published in journals, the median time to results being first publicly posted and published was 19 mo (Q1 = 14, Q3 = 30 mo) and 21 mo (Q1 = 14, Q3 = 28 mo), respectively, and the completeness of results was significantly better at ClinicalTrials.gov than in the corresponding published articles. In particular, serious adverse events were almost always reported at ClinicalTrials.gov.

A previous study assessed trial publication for completed trials registered at ClinicalTrials.gov and showed that fewer than half were published [20]. Other studies evaluated the quality of reporting of the World Health Organization minimum dataset in ClinicalTrials.gov [21]. A recent study published in 2012 [22] compared the quality of reporting among registry reports, clinical study reports submitted to regulatory authorities, and journal publications. The authors identified only industry registry reports, with no trials being registered in a public registry. They concluded that industry registry reports and journal publications insufficiently reported the results of clinical trials but may supplement each other. With the FDAAA requiring mandatory posting of results within 1 y after the primary completion date [15]–[17] and standardized reporting of results [17], ClinicalTrials.gov has become an interesting source of data for assessing trial results.

### Implications

Our results have important implications for several stakeholders: patients and clinicians, authors, researchers performing systematic reviews and meta-analyses, methodologists, peer reviewers, developers of reporting guidelines, and journal editors.

For patients and their clinicians, our results outline the importance of registries to improve transparency in clinical research by making information about clinical trials, including results, publicly available, which is the basis for well-informed decision-making about patients' health.

Our results are important for authors because they point out inconsistencies in reporting and highlight the need for more rigorous adherence to reporting guidelines to ensure that all critical information is provided in study reports.

For researchers performing systematic reviews, our results emphasize the importance of registries [23]–[27] in reducing publication bias and time-lag bias. Actually, about half of the trials with results posted at ClinicalTrials.gov did not have published results.

Further, our results highlight the need to assess trial results systematically from both ClinicalTrials.gov and the published article when available. Based on our results, searching ClinicalTrials.gov is necessary for all published and unpublished trials to obtain more complete data and to identify inconsistencies or discrepancies between the publicly posted results and the publication. As outlined by Zarin et al., ClinicalTrials.gov is designed to complement, not replace, journal publication [28]. Nevertheless, not all trials have their results posted at ClinicalTrials.gov. Some studies previously showed low compliance with the FDAAA regarding mandatory posting of results at ClinicalTrials.gov [29]–[32]. Moreover, this law concerns trials performed in the US, with no similar law in Europe or elsewhere.

Our results also highlight the role of trial registries for researchers and methodologists exploring publication bias and selective reporting of outcomes. For example, researchers could use trial registries to assess whether studies with a significant main outcome are more likely to be published or published more quickly than those with a negative outcome using data recorded in registers.

For peer reviewers, our results emphasize the important role of trial registration during the peer-review process. Actually, reviewers and academic editors could assess whether all safety events, especially serious adverse events, are fully reported in the submitted articles. In our study, serious adverse events were reported for 99% of trials at ClinicalTrials.gov but for only 62% in the published article. A study published in 2009 found that 73% of articles published in journals with a high impact factor reported serious adverse events [33]. Nevertheless, a more recent study showed that only 34% of reviewers examined information registered in a trial registry [34].

For developers of reporting guidelines, such as the CONSORT group, and for editors, our study questions the current way of reporting trials and the peer-review process. In ClinicalTrials.gov, results are posted in a standard tabular format without discussions or conclusions. Using templates with mandatory reporting of some elements may facilitate the work of researchers by reminding them what they need to report and by standardizing their reporting. Including a template for reporting the results as part of the CONSORT guidelines could be useful to improve the completeness of trials' publication.

Furthermore, after the data results are submitted, ClinicalTrials.gov staff members review the submissions before public posting [18]. Data providers may be asked to clarify items or make corrections. This systematic verification could also contribute to the completeness of data posted. These results may help convince publishers of the value of changes in the presentation of the results section of articles (standardized tabular format rather than narrative text) or of implementation of reporting guidelines. To improve the quality of reports of clinical trials, journals—even those endorsing the CONSORT statement—must move from their current position of passive endorsement (for the vast majority of them) to a more active implementation of CONSORT guidelines [35],[36].

Although the reporting of results was more complete at ClinicalTrials.gov than in the published articles, reporting at ClinicalTrials.gov is still suboptimal and could be further improved. Some elements, such as the number of patients assessed for eligibility, are nearly never reported at ClinicalTrials.gov. Other elements can be improved upon, such as the reporting of results for binary data. At ClinicalTrials.gov, the percentage of events rather than the number of events is frequently reported. Both the number of events and the number of patients analyzed per arm are needed to perform meta-analyses of binary data. Other elements raise some important issues, including the frequent reporting of nonserious adverse events observed in more than 5% of patients, 5% being the default frequency threshold for reporting nonserious adverse events at ClinicalTrials.gov according to the FDAAA [15]–[17]. The reporting of adverse events observed at a certain frequency or threshold rate has been previously outlined as poor reporting practice [37].

### Limitations

Our study has several limitations. We focused on trials with both results posted and published. It is possible that unpublished trial results could be published at a future date; some trials are submitted for publication several years after completion. When there were several publications for the same trial, we did not include all reports resulting from the trial but only the report describing the results for the primary outcomes. We chose this strategy because, according to the CONSORT statement, safety results should be reported with the main results. Only 9% of trials had multiple publications. These reports included protocols or preliminary or long-term results. None of the eliminated reports contained additional safety data for the same time frame as the selected report. Two of the eliminated reports contained additional safety results, but for longer-term follow-up. When assessing the completeness of reporting of efficacy results, we focused on a single primary outcome. If several primary outcomes were registered at ClinicalTrials.gov, we assessed the completeness of reporting for only the first primary outcome registered. Data extraction was not blinded to the source of data (ClinicalTrials.gov or published article) because blinding would have been impossible to achieve. Completeness of reporting was assessed as a binary outcome (all elements reported versus not all elements reported), so other assessment approaches may result in different findings. Finally, we could not determine whether publication, time to publication, and completeness were associated with risk of bias in trial design or conduct because ClinicalTrials.gov contained insufficient methodological information for assessing risk of bias [21],[38].

### Conclusions

In conclusion, our results highlight the importance of extracting efficacy and safety data posted at ClinicalTrials.gov not only for trials whose results are not yet published, but also for those with published results, because we found that reporting was more complete at ClinicalTrials.gov. Use of templates allowing for standardized reporting of trial results in journals or broader mandatory registration of results for all trials may help further improve transparency.

## Supporting Information

Alternative Language Abstract S1 **French translation of the abstract by AD.**
(DOCX)

Alternative Language Abstract S2 **Spanish translation of the abstract by CR.**
(DOC)

## Abbreviations

FDAAA: Food and Drug Administration Amendments Act
NCT number: ClinicalTrials.gov identification number
Q1: first quartile
Q3: third quartile
SD: standard deviation
SE: standard error
