# Supplementary material for: Timing and Completeness of Trial Results Posted at ClinicalTrials.gov and Published in Journals
Source: PLoS Med. 2013 Dec 3;10(12):e1001566. doi: 10.1371/journal.pmed.1001566 (PMC3849189; doi:10.1371/journal.pmed.1001566)
Supplement: Alternative Language Abstract S1 — French translation of the abstract by AD. (DOCX) [file pmed.1001566.s001.docx]

**RESUME (397 mots)**

**Contexte:** La Food and Drug Administration Amendments Act (FDAAA) exige que les résultats d'essais cliniques de médicaments approuvés par la FDA soient rapportés dans ClinicalTrials.gov au plus tard 1 an après la fin de l’essai.

**Objectif:** Comparer les délais entre la fin de l’essai et la date à laquelle les résultats étaient disponibles dans ClinicalTrials.gov et la date de publication ainsi que la précision des informations pour les essais avec à la fois des résultats rapportés dans ClinicalTrials.gov et publiés. **Méthodes et résultats :** Nous avons recherché en Mars 2012 tous les essais contrôlés randomisés de médicaments ayant des résultats enregistrés dans ClinicalTrials.gov. Pour un échantillon aléatoire de ces essais, nous avons recherché dans PubMed les publications correspondantes. Les données ont été extraites indépendamment dans ​​ClinicalTrials.gov et l'article publié pour les essais avec les résultats enregistrés et publiés. Nous avons comparé : 1) les délais entre la fin de l’essai et la date à laquelle les résultats étaient disponibles dans ClinicalTrials.gov et la date de publication ; 2) la précision des informations. Cette précision a été définie comme la présence de tous les éléments importants identifiés par 3 experts concernant le flux de participants, les résultats d'efficacité, les événements indésirables et les événements indésirables graves (par exemple, pour les événements indésirables, le nombre d’événements indésirables par bras, sans restriction aux différences statistiquement significatives entre les bras, pour tous les patients randomisés ou pour ceux qui ont reçu au moins une dose de traitement).

Dans l'échantillon des essais avec des résultats enregistrés dans ClinicalTrials.gov, 50% (n=297) n’étaient pas publiés. Pour les essais avec les résultats rapportés dans ClinicalTrials.gov et publiés ((n=202), le délai médian entre la fin de l’essai et la date à laquelle les résultats étaient disponibles sur ClinicalTrials.gov était de 19 mois (Q1=14-Q3=30 mois), le délai médian entre la fin de l’essai et la publication était de 21 mois (Q1=14-Q3=28 mois). Le flux des participants, les résultats d'efficacité, les événements indésirables ainsi que les événements indésirables graves étaient significativement mieux rapportés dans ClinicalTrials.gov que dans la publication.

**Limites principales de l'étude:** Nous n’avons évalué que la publication décrivant les résultats pour le critère de jugement principal.

**Conclusions:** Nos résultats soulignent la nécessité de rechercher systématique ClinicalTrials.gov pour les essais non publiés mais également pour les essais publiés. Les résultats des essais, les événements indésirables particulièrement les graves, sont mieux rapportés dans ClinicalTrials.gov que dans la publication.
