# Supplementary material for: Timing and Completeness of Trial Results Posted at ClinicalTrials.gov and Published in Journals
Source: PLoS Med. 2013 Dec 3;10(12):e1001566. doi: 10.1371/journal.pmed.1001566 (PMC3849189; doi:10.1371/journal.pmed.1001566)
Supplement: Alternative Language Abstract S2 — Spanish translation of the abstract by CR. (DOC) [file pmed.1001566.s002.doc]

**RESUMEN**

**Introducción:** La ley de enmiendas de la FDA (FDAAA), exige que los resultados de los ensayos clínicos de medicamentos aprobados por dicha agencia, sean publicados en *ClinicalTrials.gov* máximo 1 año después de su finalización.

**Objetivo:** Comparar el tiempo de publicación y la integridad de los resultados de los ensayos clínicos de medicamentos, registrados en ClinicalTrials.gov y publicados en revistas medicas.

**Métodos y Resultados:** En marzo de 2012 se realizó la búsqueda de los ensayos controlados aleatorios de medicamentos que tuvieran resultados registrados en *ClinicalTrials.gov*. Para una muestra aleatoria de estos ensayos, se realizó la búsqueda en *PubMed* de las publicaciones correspondientes. La información fue extraída de forma independiente de *ClinicalTrials.gov* y del artículo publicado. Se evaluó el tiempo transcurrido entre el desarrollo del estudio y la fecha de la primera publicación o la fecha en que los resultados se hicieron públicos, y se comparó la integridad de los resultados registrados en *ClinicalTrials.gov* con los resultados publicados en el artículo. La integridad se definió como la presentación de todos los elementos indispensables de acuerdo al consenso de 3 expertos, sobre el de flujo de participantes, los resultados de eficacia, los eventos adversos y los eventos adversos graves.

De la muestra de ensayos con resultados publicados en *ClinicalTrials.gov,* el 50% (n=297) no tenían ningún artículo publicado correspondiente. Para los ensayos con los resultados registrados y publicados (n=202), la mediana de tiempo entre la fecha de finalización del ensayo y los primeros resultados registrados fue de 19 meses (Q1=14 y Q3=30 meses) y la mediana de tiempo entre la fecha de finalización del ensayo y la publicación en una revista fue de 21 meses (Q1=14 y Q3=28 meses). El reporte de resultados fue significativamente más completo en ClinicalTrials.gov que en el artículo publicado, flujo de participantes (64% vs. 48%, p<0,001), resultados de eficacia (79% vs. 69%, p=0,02), eventos adversos (73% vs 45%, p<0,001) y eventos adversos graves (99% vs. 63%, p<0,001).

La principal limitación del estudio fue que sólo se consideró la publicación que describía los resultados del criterio primario de valoración.

**Conclusiones:** Nuestros resultados ponen en evidencia la necesidad de buscar en *ClinicalTrials.gov* tanto los ensayos no publicados como los publicados. Los resultados del ensayo clínico, los eventos adversos y especialmente los eventos adversos graves, fueron reportados de forma más completa en *ClinicalTrials.gov* que en el artículo publicado.
